# Reframing Aging: A Generation's Work

**DOI:** 10.1093/geroni/igab046.1666

**Published:** 2021-12-17

**Authors:** Patricia D'Antonio

**Affiliations:** The Gerontological Society of America, Washington, District of Columbia, United States

## Abstract

Changing American culture is challenging and changing attitudes and behaviors around the universal experience of aging especially so. Unless the field of advocates who care about aging issues cultivates a more visible, more informed conversation on older people, it will remain difficult to advance the systemic changes needed to adjust to a society with increased and increasing longevity. Advocates will need to be vigilant to avoid cueing negative attitudes towards aging and aging policies. The Reframing Aging Initiative is a long-term, social change endeavor designed to improve the public's understanding of what aging means and the many contributions older people bring to society. Using evidence-based research, the initiative seeks to teach how to tell an effective story about aging that will promote positive perceptions of aging and reduce ageism.

